# Differentiated experiences of financial precarity and lived precariousness among international students in Australia

**DOI:** 10.1007/s10734-023-01033-9

**Published:** 2023-05-13

**Authors:** Benjamin Mulvey, Alan Morris, Luke Ashton

**Affiliations:** 1 School of Education, University of Glasgow, Glasgow, G12 8QQ UK; 2 Institute for Public Policy and Governance, University of Technology Sydney, 15 Broadway, Ultimo, NSW 2007 Australia

**Keywords:** International student mobility, Precarity, Housing, Work, Migration

## Abstract

Empirical research on international student migrants has sometimes homogenised this group, framing it as predominantly made up of privileged members of the global middle-class. This has led to calls to acknowledge and address the precarity faced by international students in their respective host countries more comprehensively. This study aims to explore how levels of financial precarity vary among international students in Australia, and how this in turn contributes to varying levels of precariousness in the personal spheres of students’ lives. In doing so, we centre and refine the concept of precarity for use in studies of internationally mobile students, arguing for its use as a ‘relational nexus’, bridging financial precarity and broader lived experiences. Drawing on a large-scale survey and semi-structured interviews with 48 students, we emphasise the linkages between financial precarity and precariousness as a socio-ontological experience, explored through the examples of time poverty, physical and mental wellbeing, and relationships.

## Introduction

The number of international students in Australia has expanded rapidly over the past three decades, in line with a trend towards global expansion in the number of globally mobile post-secondary students: there were more than 5.3 million people studying for a tertiary qualification outside of their home country as of 2017 (UNESCO, 2021). Australia has benefitted from this migratory flow, having developed a highly lucrative higher education export industry. In 2021, despite the COVID-19 pandemic, there were 570,626 students enrolled in Australian tertiary institutions, making it the fourth most popular destination country for globally mobile students (DoE, 2022).

These international students are generally framed as a relatively homogenous group, through consumption-based metaphors, as privileged members of the ‘global middle class(es)’ (Robertson, 2015). This is reflected in the fact that a significant portion of the empirical research on internationally mobile students has been concerned with the ways in which this form of mobility is employed as a means of middle-class social reproduction (e.g. Waters and Brooks, 2021; Lee, 2022; Mulvey, 2021). As a result, research in this area, especially that focused on major destinations in the Global North, has only rarely acknowledged the full diversity of socio-economic backgrounds within this group. However, there is increasing recognition of a critical need to acknowledge the vulnerability and precarity faced by many international students, progressing beyond the aforementioned ‘flattening’ of the socio-economic dimensions of international student mobility (Gilmartin et al., 2021; Lipura & Collins, 2020; Raghuram et al., 2020; Morris et al., 2022a).

In this article, based on data from a mixed-methods study, we seek first, to explore how levels of financial precarity vary among international students, and second, to investigate the ways in which financial precarity seeps into the ‘intimate spheres’ of international students’ lives. In doing so, an aim is to centre and refine ‘precarity’ as a conceptual framing for the study of international student mobility. We posit its use as a bridge or ‘relational nexus’ (Neilson, 2019), anchored in structural conditions but connecting these to the broader experiences of students, focusing on time poverty, social isolation, and negative impacts on physical and mental wellbeing, as facets of precariousness understood as ‘a socio-ontological dimension of lives and bodies’ (Lorey, 2015, p.11). In other words, rather than viewing precarity narrowly as an exclusively economic condition on one hand, or as an overly broad ontological category on the other, we seek to highlight the relationship between financial precarity and facets of lived experience (Millar, 2017; Strauss, 2018) . To do so, we draw on survey and interview data to explore the mechanisms through which financial precarity contributes to precarity of lived experience among international students.

The findings that we present in this paper contribute to understandings of precarity among international students in two ways. First, we highlight the high level of variation within this group, drawing on the results of a survey of over 7000 international students in the private rental sector in Sydney and Melbourne, Australia’s two largest cities. The survey collected information on the number of financial stress indicators students experienced in the last year and premised on the number experienced students are divided into four precarity groups—secure, moderately precarious, highly precarious, and extremely precarious (Morris et al., 2022a). Second, the division of students according to levels of financial deprivation enables an exploration of the ways in which economic insecurity, and other facets of broader lived experience—wellbeing, free time, and relationships—interact with each other and shape migrants’ experiences differentially along lines of financial vulnerability.

The next section of the paper outlines the Australian policy context—an immigration regime that contributes to the creation of conditions of precarity for some temporary migrants including international students—before reviewing the existing literature on international student precarity. The third section develops a framework for understanding the interactions between precarity as a socio-economic condition, and precariousness as a broader lived experience. Following this, we outline the methodology and detail the findings.

## Precarity among international students in Australia

Australia has developed a highly successful higher education ‘export industry’ over the past several decades and is among the most popular destination countries for internationally mobile tertiary students (UNESCO, 2021). International students in Australia are a crucial source of institutional revenue for the post-secondary sector, contributing approximately 40 billion dollars per year (ABS, 2022). This means that international education is Australia’s largest service sector export. As of 2018, the majority (46%) of tertiary students in the country were enrolled as university students, with others enrolled either in vocational education and training courses (30%) or English language courses (16%). The remaining 8% are enrolled in non-award programmes or in schools (Australian Government, 2019).

A significant portion of extant research has emphasised that international student mobility, in Australia and other destination countries, is an overwhelmingly ‘middle-class’ activity (e.g. Waters and Brooks, 2021; Lee, 2022). In other words, student-migrants are often understood through what Robertson (2015, p. 941) describes as ‘consumption-based metaphors of privileged mobility’. However, this is a one-dimensional and incomplete framing of the experiences of international students, who are not invariably privileged, and who may experience precarity in various domains (Lipura & Collins, 2020). Chacko (2021), for example, highlights how immigration regimes create precarity for international students in Singapore, and Gilmartin et al. (2021) explore the experiences of precariousness of international students in The Republic of Ireland.

There is a nascent body of work focused on the Australian context which sheds light on the various forms of exploitation and vulnerability experienced by international students. This research tends to emphasise financial precarity through a focus on employment and housing conditions (Nyland et al., 2009; Berg & Farbenblum, 2019; Clibborn, 2021; Reilly et al., 2020). Taken together, this research constitutes a body of evidence that demonstrates the governmental precarisation (Lorey, 2015) of international students in Australia. In other words, it shows how economic precarity among international students in Australia is politically and institutionally produced. This is achieved in two main ways. First, the limit on the number of hours students are allowed to work contributes to the systemic underpayment of international students who undertake employment. Approximately half of the respondents in Berg and Farbenblum’s (2019) survey reported being paid less than the statutory minimum wage. Second, international students are not seen as rights-bearing subjects of education of welfare systems, meaning they do not have access to many of the welfare protections afforded to local students, such as income assistance and rental subsidies, and must also pay tuition fees at a much higher rate (Marginson et al., 2010; Hastings et al., 2021).

In addition to exploitation in the workplace, a number of studies have examined how the financial precarity of international students also translates into housing stress (Berg & Farbenblum, 2019; Ruming & Dowling, 2017; Morris et al., 2021 Morris et al., 2022b). Over a decade ago, Marginson et al. (2010) highlighted that the provision of international student housing in Australia was in ‘crisis’, and the situation appears not to have improved in the intervening years. The Australian Bureau of Statistics (2013) found that international students, and particularly those from low-income countries, were more likely to live in overcrowded accommodation. Berg and Farbenblum (2019) and Morris et al. (2021) also found that half of the respondents to their survey were experiencing at least one problem with their accommodation, and Ruming and Dowling (2017) present evidence that negative experiences of both securing accommodation and of housing quality were the norm among a small group of international postgraduate students in Sydney. Other work from this project has highlighted that housing-related problems such as rental affordability and overcrowding are significant issues for international students (Morris et al., 2022b; Hastings et al., 2021).

This increasing recognition of the precarity experienced by predominantly ‘middle-class’ international student migrants in Australia demonstrates a flaw in sociological axioms around the relationship between mobility and privilege. Bauman (1998, p. 86) for example contrasts the freedom and autonomy of privileged members of society to be mobile with the restricted mobility of those less fortunate:

the dimension along which those ‘high up’ and ‘low down’ are plotted in a society of consumers, is their degree of mobility – their freedom to choose where to be.

In other words, those at the top of the social hierarchy move with ease, whereas those at the bottom have little freedom of choice over mobility, and are ‘pushed from behind’. But on the other hand, as Robertson et al., 2022, p. 2) highlight, the mobility of relatively privileged middle-class subjects, a choice in itself, has the potential—for some—to produce a lack of freedom of choice in other domains, as exemplified by the multiple forms of precarity that we seek to explore here:

to be on the move, even as a privileged subject, has become arguably more precarious, and the relationship between social and spatial mobility remains, in practice, complex and unevenly experienced by different groups within the broad spectrum of ‘the middle’… Young people from middle class backgrounds face increasing economic insecurity and unstable life course trajectories, a condition that can be both ameliorated and amplified by transnational mobility.

In this vein, existing studies highlight economic precarity among international students, focusing on workplace precarity (e.g. Berg & Farbenblum, 2017; 2019; Clibborn, 2021; Reilly et al., 2020; Wilson et al., 2022) and housing stress (Berg and Farbenblum, 2019; Morris et al., 2021; Ruming & Dowling, 2017).

In this paper, we offer a more fine-grained approach to understanding the nature of the precarity by highlighting the highly varied nature of precarity between groups of students, categorised according to the level of financial stress they have experienced in the last year. Second, we move understandings of the precarity of international students—identified as a critically important yet neglected avenue for investigation (Lipura & Collins, 2020; Raghuram et al., 2020)—forward in two ways. We advocate a relational approach to the use of precarity as an analytical tool, as well as emphasising the linkages between economic precarity and the broader lived experiences of international students.

## Conceptual framework

Here, we advocate a particular understanding of precarity as a bridge between economic conditions and lived experiences. Shierup and Jorgensen (2016, p. 949) posit that the ‘migrant’ is the ‘quintessential incarnation of precarity’, as for migrants the precarisation of labour operates concurrently with the splintering of frameworks of citizenship in the Global North. Clearly, these sources of precarity shape the experiences of international student migrants, too. Hastings et al. (2021) for example explore the implications of this combination for international students’ personal finances in Australia.

The term ‘precarity’ is used widely, including in studies of international student mobility (Cairns et al., 2021; Chacko, 2021; Gilmartin et al., 2021; Lipura & Collins, 2020; Raghuram et al., 2020). However, this usage often lacks conceptual clarity, employed as if it is synonymous with insecurity or vulnerability, but this is an issue because, as Millar (2017) observes, to conflate precarity with other phenomenon in this way weakens its analytical power through dilution. In this paper, an aim is to integrate the literature on international student migrants, as a distinct sub-category of transient migrants, with broader debates around the definition of precarity. Here, we delineate how the term has been used in the academic literature, and how it is used in this paper as an analytical concept.

Conceptual understandings of precarity can, broadly speaking, be divided into three groups (Millar, 2017). First, the dominant view of precarity as an economic phenomenon—either purely as a labour condition or as a social class grouping. Bourdieu (1963) was among the first sociologists to explore the concept of precarity in his early work on underemployed Algerian workers. Guy Standing (e.g. 2018) then popularised the term ‘precariat’, arguing that this group represent a broad ‘class-in-the-making’, defined by its lack of labour security, stable, consistent income, and strong work-based identity.

A third body of literature takes a much broader definition of precarity as an ‘ontological experience of human existence’ (Millar, 2017, p. 2) or, in other words, ‘a feature of broader life’ (Lewis et al., 2015, p. 584). From this perspective, precarity is a socio-economic condition, but crucially it is also ‘something more’ than this (Neilson & Rossiter, 2005). Research in this vein tends to explore how economic precarity is inevitably entangled with other areas of life. Lorey (2015, p. 11) for example explores how governments produce both precarity and precariousness, or ‘a socio-ontological dimension of lives and bodies’. We emphasise the utility of highlighting the links between economic precarity and precarious lives more broadly writ. This relational approach to precarity, providing a bridge between a critique of political-economic structures which anchors the term precarity, and questions of lived experience of precariousness (Mallett, 2020), has value here in understanding how economic precarity intersects with and contributes to precariousness as Lorey (2015) describes it in the case of international student migrants.

As such, we draw on a definition of precarity which ‘emerges at the crossroads of individual experiences and structural conditions’ (Dotsey & Chiodelli, 2021, p. 732). In other words, we seek to examine how precarity, in the economic sense, leads to precariousness, in the sense of a lived experience. This enables a recognition that the ways in which financial precarity impacts the lives of student migrants must be understood within an overarching framework of insecurity that affects all aspects of life. To do this, we first categorise survey respondents according to their level of financial deprivation, before examining how precarity shapes the lives of international students—differentially along the lines of levels of social privilege—in two domains work and housing. We then explore the implications of financial precarity for ‘precariousness’ (Lorey, 2015) in broader lived experiences of participants. We develop a framework of ‘multiple intersecting precarities’, identifying a number of interrelated focal points for analysis related to the personal spheres of students’ lives: the examples of housing, work, time poverty, wellbeing, and relationships. Understanding the linkages between these spheres enables a rich understanding of the difficulties faced by international students depending on their level of precarity.

## Methodology

The data used in this paper was collected as part of a mixed methods project focused on housing precarity among international students in Australia’s private rental sector. We draw on an initial survey conducted between August and December 2019 in Sydney and Melbourne, and semi-structured interviews conducted with 48 international students. The survey focused on students’ experiences of the private rental sector and also included items related to student wellbeing, social capital, and a range of indicators of financial stress.

Tertiary education institutions in Sydney and Melbourne were approached and asked if they would send a link to the survey to all of their international students. Ten universities, 24 vocational education providers, 7 English language colleges, and 2 foundation colleges agreed to support the study and a total of 7084 valid responses were received. The survey was available in either English or Mandarin Chinese in order to ensure a high response rate among Chinese students, as China represented the largest source country of international students in Australia at the time the survey was conducted.

This data is supplemented by insights from 48 in-depth semi-structured interviews. The interviews covered a wide range of themes including friendship and social ties, loneliness, paid employment, financial stress, finding accommodation, housing insecurity, and housing quality. They were conducted via Zoom, as they were undertaken during the pandemic when face-to-face meetings were not possible. A shortlist of 120 respondents to the survey who indicated a willingness to be interviewed in-depth was developed based on the composite precarity score, with an aim of selecting students with a wide variation of experiences of precarity. The interviews were analysed through both deductive and inductive coding, using NVivo qualitative data analysis software. Through the coding, the research team drew out key findings regarding the financial insecurity and students’ lived experiences. In particular, the selected interview excerpts were chosen to underline the potential mechanisms, identified through the coding process, through which financial insecurity led to differential outcomes in terms of the other forms of precariousness, highlighted by the survey.

### Defining financial stress

Central to this article is an understanding of students’ experiences of financial stress in Australia, a country ranked as among the most expensive to live in for international students (HSBC, 2013). A modified version of the Australian Bureau of Statistics’ (ABS) financial stress questionnaire was employed in order to capture levels of financial stress experienced by students within the sample. Of the nine indicators used in the ABS questionnaire, two were removed: ‘household spends more than it gets’, and ‘unable to raise Australian $2000 in a week for something important’. These items were deemed to have little applicability to the situation of international students and were replaced with one new item which asked about the affordability of textbooks.

**Fig. 1:**
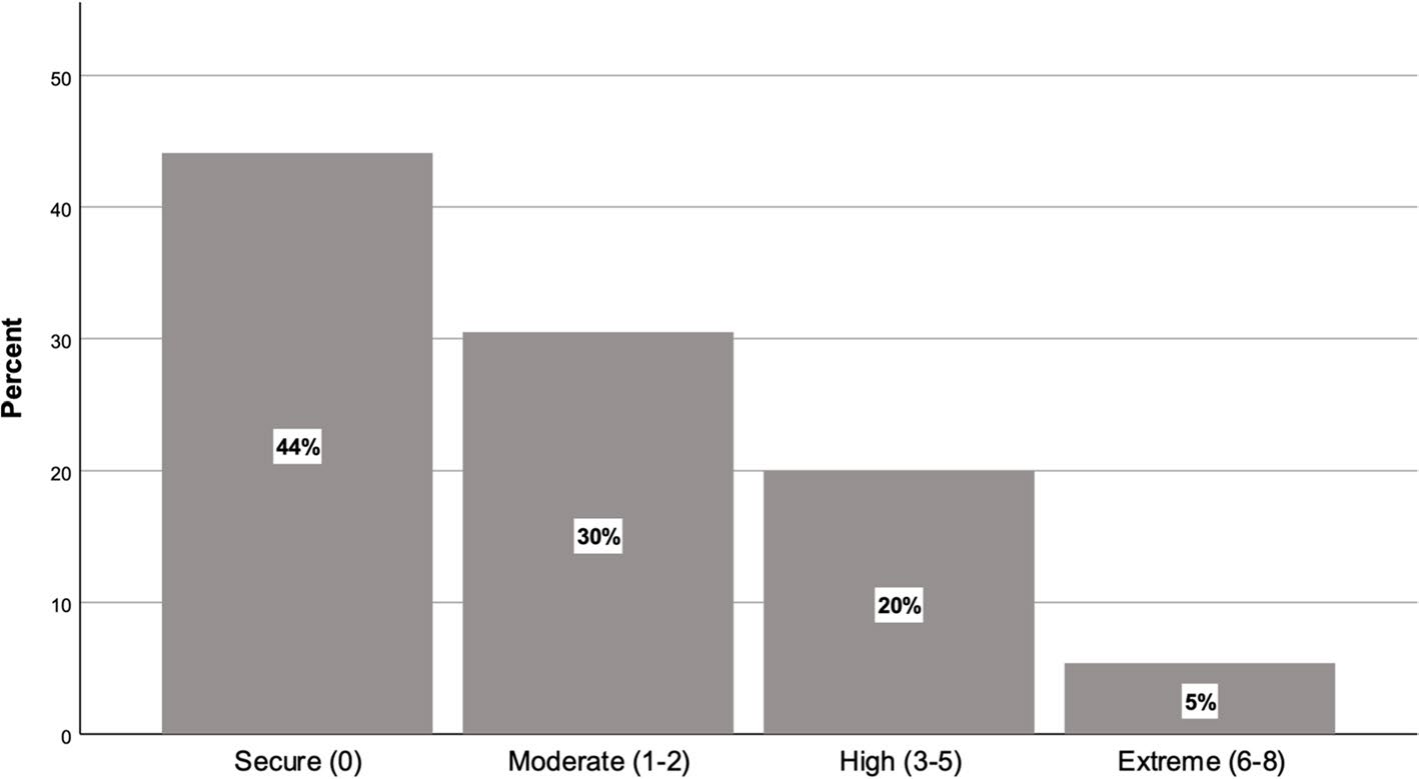
Four groups of financial stress in 2019

In order to ascertain the overall level of financial stress experienced by respondents, the number of financial stress items individual respondents indicated they experienced in the last 12 months was aggregated into a score between 0 and 8. Figure 1 shows the distribution of these scores—around 55% of the students that responded to our survey in 2019 reported experiencing at least one instance of financial stress.

Principal component analysis (PCA) was used to identify latent factors in the data, without having to employ all eight variables in our analysis. Meaningful factors were identified using the total percent variance explained approach. In this analysis, three factors explained most of the variance across the set of eight variables. These factors, and the variables which constitute them, formed cut-off points for the four separate groups of students. The distribution of respondents into these four groups is shown in Fig. 1. Those who did not report instances of financial stress constitute the first group. Those who reported 1 to 2 instances of stress are referred to as ‘moderately’ precarious, 3 to 5 as ‘highly’ precarious, and 6 to 8 as ‘extremely’ precarious. Throughout the findings and discussion section, all figures using these four groups of precarity were tested for significance using both Fisher’s exact test and Pearson’s chi-squared test and were found to be significant at the 95% confidence level.

## Findings and discussion

In this section, we seek to establish how the level of financial precarity students experience shapes broader lived experiences in the host country. We examine the ways in which financial stress translates into experiences of work and housing in Australia. Employing survey data, supplemented by interview data, we demonstrate that international students differentially experience precarity in work and precarity of place in Australia, according to their level of financial stress. We then explore how levels of precarity in these domains, shaped by financial stress, in turn contribute to personal precariousness, focusing on time poverty, general health and wellbeing, and personal relationships as facets of this. While the findings and discussion are separated into these key themes, there is inevitably some overlap between them, as they are mutually reinforcing.

### Precarious work

**Fig. 2:**
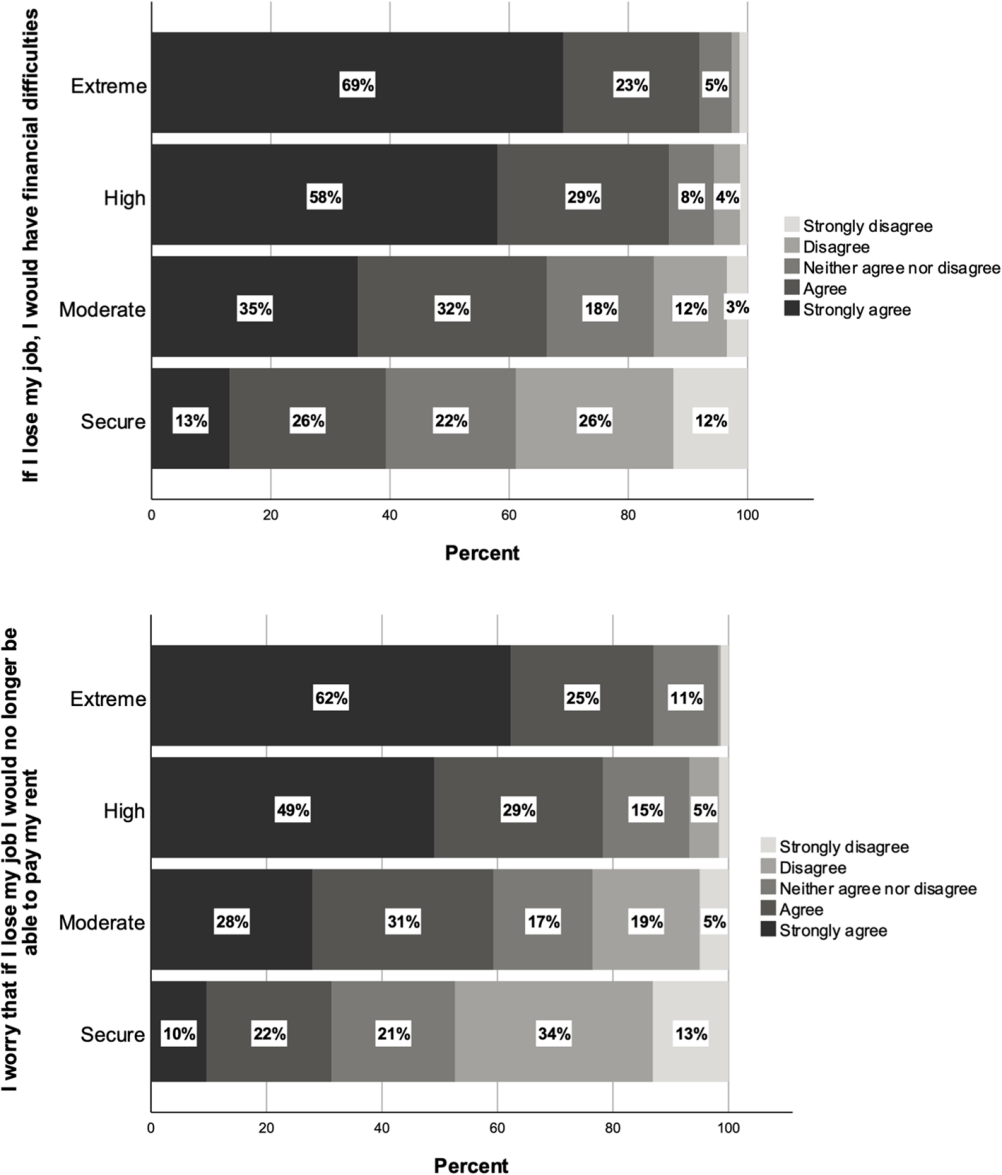
Responses to survey items: ‘If I lose my job, I would have financial difficulties’ and ‘I worry that if I lost my job I would no longer be able to pay my rent’

Our survey data highlight that students experiencing higher levels of financial precarity were more likely to see work as a necessity. While this seems self-evident, after establishing this, we seek to emphasie the broader implications for students’ experiences in Australia. Figure 2 shows responses to two survey items. The first item shows the substantial differences in responses to the survey question ‘If I lose my job, I would have financial difficulties’ among the four financial precarity groups. Ninety-one percent of extremely financially precarious students agreed with this statement, and of these 69% strongly agreed. In the highly precarious group, 87% agreed with this statement, compared to 67% in the moderately precarious group, and 39% in the financially secure group. The second item in Fig. 2 similarly shows a powerful association between financial precarity and the need to work. Eighty-seven percent of the extremely precarious and 78% of highly precarious agreed with the statement ‘I worry that if I lost my job I would no longer be able to pay my rent’, compared to 59% of the moderately precarious and 32% of those not experiencing financial precarity.

### Precarious housing

**Fig. 3:**
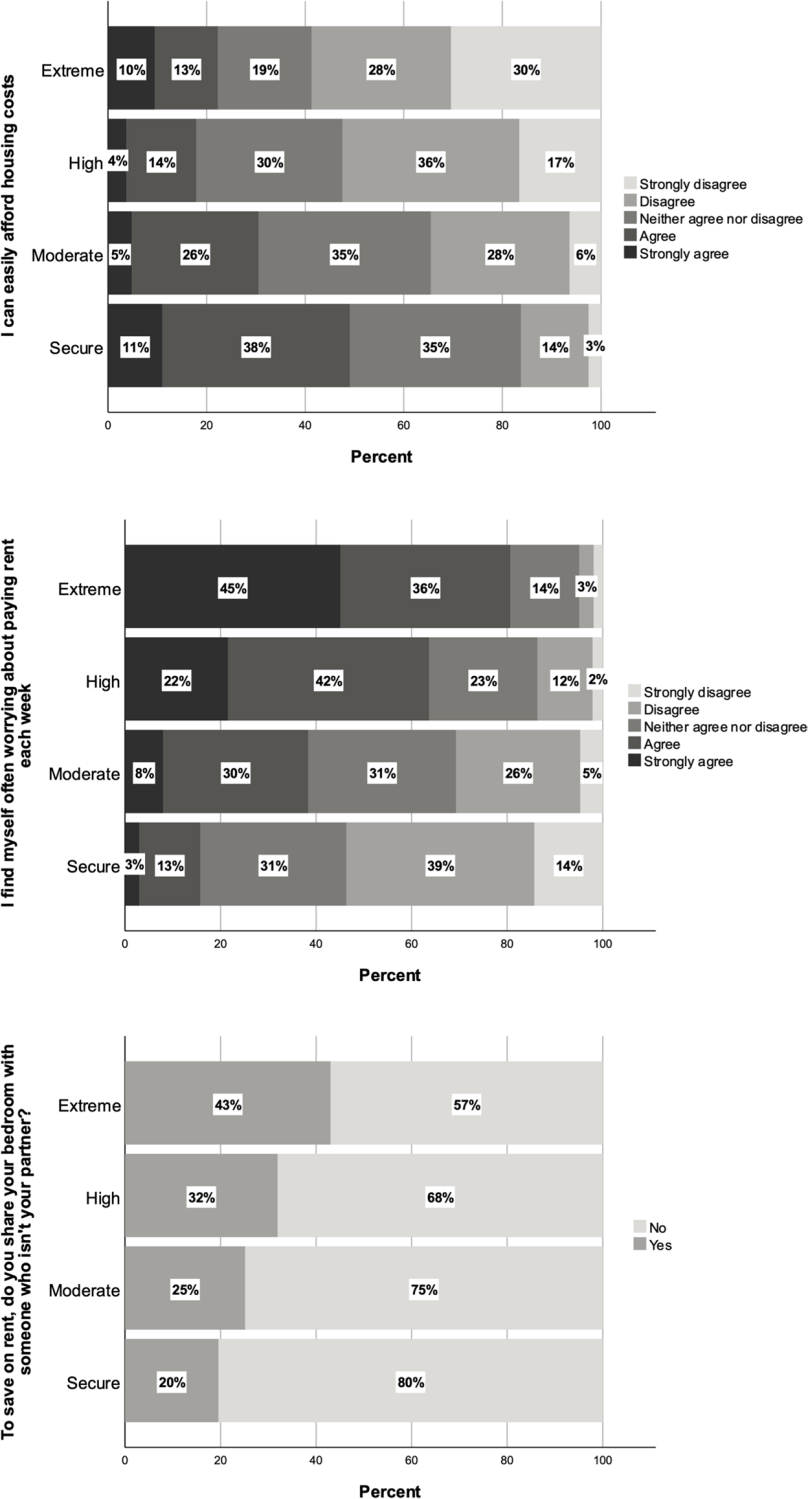
Responses to survey items: ‘I can easily afford housing costs’, ‘I find myself often worrying about paying rent each week’, and ‘To save on rent, do you share your bedroom with someone who isn’t your partner?’

Another clear trend in the data is that students who experienced higher levels financial stress were more likely to suggest through survey responses that they had difficulty covering housing costs in Australia, and worried about this often. Figure 3 shows the distribution of responses to the statement ‘I can easily afford housing costs’ by financial stress group. Fifty-nine percent of extremely precarious students and 52% of highly precarious students disagreed with the statement, whereas only 16% of those not experiencing financial stress shared this sentiment.

The figure also suggests that ability to afford housing costs has an impact on students’ wellbeing: 81% of the extremely precarious group reported that they worried about paying rent each week, compared to 64% of the highly precarious, 38% of the moderately precarious, and just 16% of those not experiencing financial insecurity. As Fig. 3 shows, these students were also more likely to share rooms with others in order to reduce rent burden. Forty-three percent of extremely precarious students shared their bedrooms with a non-partner, compared to 32% of highly precarious students, a quarter of moderately stressed and one in five of those in the secure group.

Those in the extremely precarious group were also more likely to indicate that they have to ‘hotbed’, that is, to share a bed that is only available for a few hours of the day or night. 13 percent of extremely precarious students answered yes to this question. These findings highlight significant differences between international students in terms of their ability to afford housing. Difficulties in affording housing mean that those within the extremely and highly precarious groups are more likely to worry about paying rent and more likely to live in conditions that have the potential to contribute to insecurity and vulnerability in other domains, as we explore in the following section.

### Precarious lives

In this section, we present and discuss findings which indicate mechanisms through which the two factors outlined above shape students’ broader lived experiences. In other words, we move the discussion forward towards an understanding of the ways in which financial precarity produces precariousness in the broader lived experiences of international students. The purpose is to demonstrate why the work precarity and housing affordability stress outlined above must be understood within a broader framework of insecurity. We focus on the impact of financial stress on time poverty, health and wellbeing, and the development of personal relationships.

### Time poverty

**Fig. 4:**
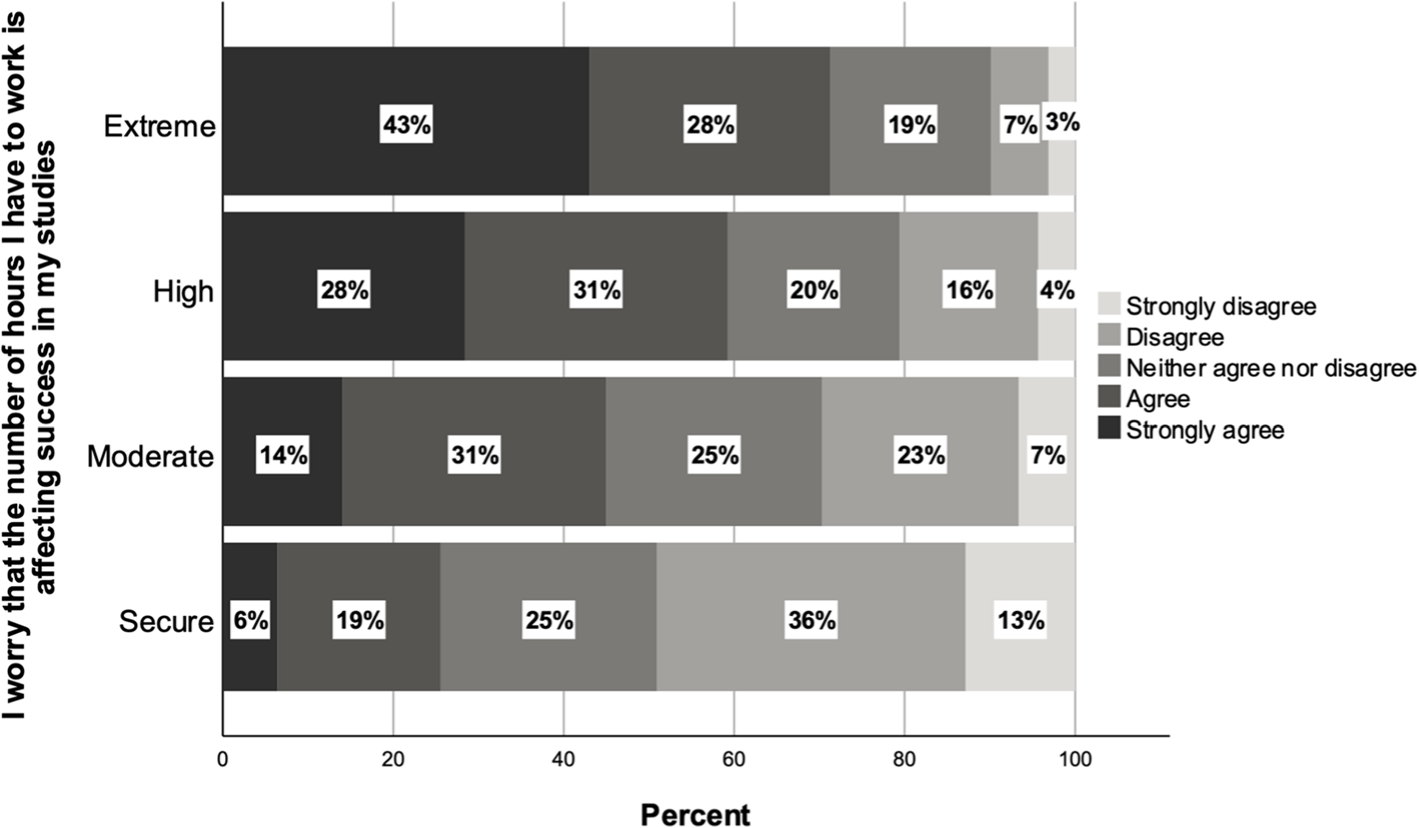
Responses to survey item: ‘I worry that the number of hours I have to work is affecting success in my studies’ (*n* = 2873)

Students experiencing high and extreme levels of financial precarity were more likely to agree with the statement in Fig. 4: ‘I worry that the number of hours I have to work is affecting success in my studies’. This highlights the linkage between financial precarity and time poverty. Seventy-one percent of students in the extremely precarious group agreed with this statement, compared to 59% of the highly precarious, 45% of the moderately precarious, and just 25% of the financially secure. Interview data highlights the mechanisms through which financial precarity and the resulting time poverty contributed to feelings of anxiety, stress, and more broadly, the negative impacts on health, wellbeing, and the development of personal relationships that we explore further in the following sections. An example is that of Zayan, a bachelor’s degree student from India, studying in Sydney. She describes her work routine in the months after her arrival in Australia:

I had literally done three jobs… one a barista, one at a restaurant, then another cleaning. Just to manage my fees and somehow literally have some money, just so I can pay rent afterwards and all that stuff. Then doing the night shifts, afternoon, mornings everything at that time… So I left the cleaning job at that time because that was the most tiring one and they wanted the night shifts to be done. But it was from 9pm until 4am in the morning… after that I would come back and at 5.30 I would go to the barista one, and then in the evening there was the restaurant one, so the whole day like getting two or three hours of sleep was not enough

This routine ultimately impacted on Zayan’s physical health:

I wasn’t able to sleep well, I wasn’t eating well, everything actually had a disadvantage on my body… last year I had to go to the hospital to get surgery done, because all of these things I had done to my body.

It is also worth noting that Zayan’s need to work multiple jobs was partly due to wage theft by some of her employers. She was paid just Australian $10 per hour for the first position she found, as a cleaner in the hospitality sector. This was around half of the basic statutory minimum wage, which was Australian $19.49 when the interview took place. Wage theft is a widespread phenomenon in Australia. Farbenblum and Berg (2019, p. 8) found that underpayment of international students is ‘systemic and widespread’, with 49% of respondents to their survey reporting being paid below the basic statutory minimum wage, and 77% being paid below the minimum casual hourly wage. Overall, Zayan’s case highlights one pathway through which financial precarity seeps into other spheres of students’ lives, such as the development of social networks, and broader health and wellbeing.

Another example is that of Clara, studying for a vocational qualification in Melbourne. She outlines her struggles balancing work and study, and describes how this led to the onset of anxiety and depression:

I got really stressed and anxious about my grades as well, because I consider myself a very organised person, and at the same time, I always aim for the best. So, when I arrived here I have this mentality that I know that’s going to be hard, because it’s way different that I have to work part-time, and then have to study full-time… So that mentality that I have to work and then the units that I took when I arrived here was really too much for me… so I was like, “Oh my god, how will I pass this?” That’s the level of my anxiety and my depression during that time. My university offers a health and wellbeing set-up where you can talk to a psychologist, so yeah I had a few sessions, actually I guess, if I'm not mistaken, I had six sessions with the psychologist, just for me to really get over the feeling, and yeah, the depression.

Overall, findings from the interviews highlight how financial precarity often necessitates work, which for international students in Australia is often both insecure and underpaid (Clibborn, 2021; Farbenblum & Beck, 2020). This inevitably led to time poverty among many of the most financially precarious students, and this time poverty in turn has potential implications beyond work and housing, contributing to broader precariousness for some international students. A number of existing studies have explored the effects of time poverty in tertiary education, highlighting how the need to work has broader negative implications for the experiences of students (e.g.Burston, 2017; Robotham & Julian, 2006; Rubin & Wright, 2017). As we explore in the following sections, time poverty represents one mechanism through which financial precarity contributes to negative impacts on physical and psychological wellbeing and the development of relationships.

#### Physical and psychological wellbeing

**Fig. 5:**
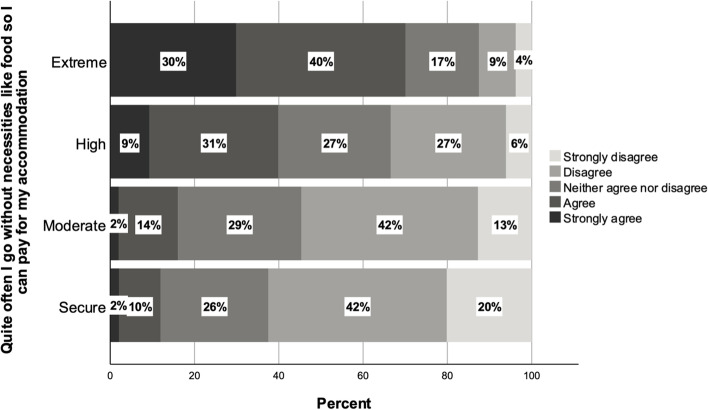
Responses to survey item: ‘Quite often I go without necessities like food so I can pay for my accomodation’ (*n* = 6089)

Central to our analysis is the notion that financial precarity must be understood as part of an overarching framework of insecurity. The conception of precarity is anchored in economic conditions, but economic precarity creates precariousness in various other domains (Lorey, 2015). The cases of both Zayan and Clara, highlighted in the previous section, underline how financial precarity leads to time poverty, but also, how it may shape broader wellbeing. Figure 5 shows responses to the statement: ‘Quite often I go without necessities like food so I can pay for my accommodation’. This survey item is chosen as it highlights one mechanism through which financial precarity may negatively impact students’ general psychological and physical wellbeing. Again, there are clear differences between those in situations of ‘extreme’ financial precarity and others: around 70% of students in this category reported regularly going without basic necessities such as food in order to pay rent, compared with 40% of the highly precarious, 16% of the moderately precarious, and 12% of those not experiencing financial stress.

Qualitative data sheds some light on the ways in which high levels of financial precarity shaped the wellbeing of international students. In particular, covering rental costs was a source of unease and anxiety for students. Ayaan is an undergraduate international student from India, studying engineering at an institution in Melbourne who experienced financial difficulties throughout his stay in Australia. He was heavily reliant on part-time casual work, but had struggled to find and keep jobs consistently. He describes his experience of having to skip meals in order to pay rent during the months after his arrival in Australia:

It’s just like you have this thing in your mind that you have to pay somebody back, you have to save up for your rent and your fees I think that was the time when you have to put it all off. So we have seen the days where we wouldn’t [be] eating all day and that actually also affected me physically, not eating [the] whole day and relying on one meal and just water.

As the survey data suggest, this was a relatively common experience among respondents experiencing significant financial stress. Students such as Ayaan described how economic precarity among international students has the potential to disrupt other aspects of everyday life.

In other cases, the experience of ‘going without’ due to financial stress directly impacted health, insofar as it motivated students to avoid seeking medical treatment. Fatemeh, a PhD student in Sydney, described how, as a full-time student, she is reliant on her husband’s income from employment. This caused problems because her husband’s salary was, as she describes: ‘very low, like $2,000 per month some months, and some months nothing’. The experience of a close family member passing away, combined with her financial precarity, contributed to Fatemeh being diagnosed with depression. In the following excerpt, Fatemeh explains how her financial situation made her reluctant to persist with therapy:

Last year after that trauma, that depression that I had, my GP in university emphasised that I should go to a therapist. I went to three sessions but after that, even those three sessions I was thinking, why am I paying this money? … I was depressed, but at the same time there is a joke that goes: when you go to therapist and pay that money, you get more depressed.

She also mentioned that paying the rent on time sometimes meant foregoing other important expenses such as dental care:

Me and my husband and some of my lab mates, for more than two years we didn’t go to dentist. We need to go, but we can’t go.

These examples demonstrate another mechanism through which economic precarity contributes to precariousness in students’ lived experiences. Specifically, the evidence presented highlights how insecure employment and housing affordability stress creates mutually mutually reinforcing precarities for less affluent international students. Financial stress contributes to a sense of anxiety, unease, and in some cases, to negative impacts on mental and physical health. Forbes-Mewett and Sawyer (2019) highlight that international students are particularly vulnerable to mental health problems, which are often exacerbated by the stress associated with living in a foreign country. We emphasise that not all international students are equally vulnerable: the most financially insecure appear to be more susceptible in this regard.

Diminished ability to develop social networks due to time poverty is also likely to further aggravate these issues, as we explore in the following section. This aligns with the Alharbi and Smith’s (2019) observation that time management and the ability to unwind after studying are important factors predicting the overall wellbeing of international students. This highlights one pathway through which time poverty may contribute to worsened overall wellbeing, in turn, further aggravating precariousness.

#### Relationships

**Fig. 6:**
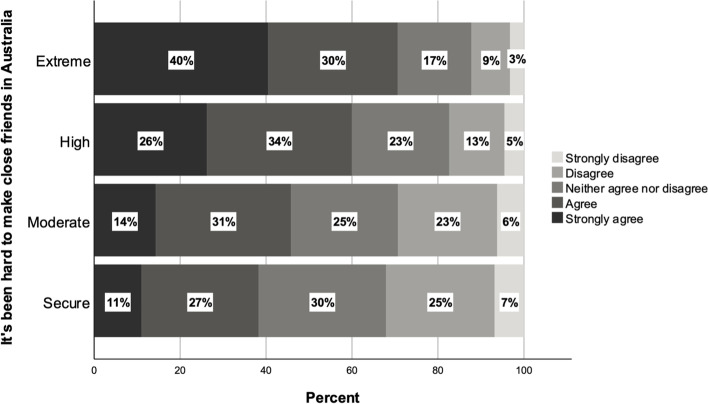
Responses to survey item: ‘It has been hard to make close friends in Australia’ (*n* = 6816)

Precarity and financial stress are often associated with social isolation and individualisation (e.g. Lorey, 2015; Mallett, 2020; Morris et al., 2022b). Our findings highlight how the work and housing conditions of the students experiencing the highest levels of financial stress may contribute to social isolation, one factor in the broader insecurity that the most financially precarious experience. Those in the extreme and high precarity groups were more likely to report difficulties in developing close friendships: Fig. 6 shows that those in extreme financial precarity were almost two times more likely than financially secure students to agree with the survey item: ‘It’s been hard to make close friends in Australia’. Seventy percent of extremely precarious and 60% of highly precarious students agreed with this statement, compared to 45% of the moderately precarious and 38% of the financially secure. Sara, a postgraduate nursing student living in Sydney, described how her housing situation contributes to her social isolation and difficulties in making friends:

I’ve lived away from my family for six or seven years already, because I never stayed in my own city. It was, I always had a different kind of place that I lived with, all of the people sitting together, watching movies… So when I compare it with that, I do feel that this place, maybe it’s my housing or whatever in general in Australia, it’s really isolated. Everyone just wants to stay in their room, which I do appreciate because I want that as well, but you know, sometimes you want a bit of outlet.

In this case, a lack of communication with housemates left Sara feeling lonely and isolated. The experience of living in a house with a large number of occupants but little sense of connection or communication between them intensified this feeling (see Morris et al., 2022b). A similar case is that of Pranit, an international student from India, studying an undergraduate degree in nursing in Melbourne. He lived in shared accommodation with four others, sharing a room with another student in order to save on rent. Like Sara, Pranit felt that living in a crowded house with housemates—all of whom applied independently for a space in the house—intensified feelings of isolation. He suggests that living in crowded accommodation did not alleviate feelings of loneliness: ‘even if you’re surrounded by so many people still you can be lonely’. He felt that was primarily because of a lack of connection with his housemates:

I just think differently from the way they do … They’re good in their own way but we don’t just get along very much. We are friends, but we don’t get along very much… we do stuff together but still, we aren’t connected that much personally.

This evidence of difficulty making social connections and maintaining ties with others while studying in Australia highlights further the ways in which financial precarity contributes to precariousness in broader lived experiences. Economic precarity can produce social isolation for a number of reasons (Lorey, 2015), and this is especially true for migrants who are already isolated from families and other support systems (Lewis et al., 2015) . Sawir et al. (2008) highlight that social isolation among international students is a common problem, most obviously because of the loss of contact with families and broader social networks in the home country. But beyond this, our findings suggest that students experiencing a greater level of financial stress may have more difficulty in this regard than their more financially secure counterparts.

The forms of precarity highlighted here are interlinked, and both time poverty and housing affordability stress appear to have contributed to both Sara and Pranit’s social isolation. To expand on this, one contributory factor in these cases appears to be the particular housing situation these students found themselves in. Students experiencing greater levels of financial precarity were more likely to live in overcrowded accommodation and more likely to share rooms or even ‘hotbed’. As well as contributing to feelings of vulnerability (Green & McCarthy, 2015), this lack of personal space can, counterintuitively, act as a barrier to the creation of social connections with other sharers (Heath, 2004). This is in part because of a lack of common space in such households, which discourages meaningful interaction and the development of strong ties between those sharing the space (Heath et al., 2017) . Moreover, as we explore in other publications resulting from this project (Morris et al., 2022b), a lack of personal space to withdraw to may actually discourage students from seeking connections with housemates.

Furthermore, the greater need to undertake employment, as highlighted by the relatively high proportion of extremely precarious students expressing anxiety about the number of hours worked, represents another mechanism through which financial precarity and students’ existential state are intertwined. The need to work in addition to attending their course of study inevitably reduces opportunities to socialise both formally, through clubs and societies for example, and informally. These activities play an important role in reducing social isolation among international students (e.g. Elliot et al., 2016).

Just as the temporal facet of precariousness also contributes to the social, there is increasing evidence of the impact of loneliness, or in other words of the social dimension of precariousness, on psychological and physical wellbeing. Richardson et al. (2017) for example find that greater levels of reported loneliness predict greater levels of stress, anxiety, and depression among a group of British undergraduate students, and Holt-Lunstad et al. (2010), based on a meta-analysis of 148 studies, suggest that the impact of loneliness on risk of death is comparable to that of obesity and excessive smoking. Similarly, Alharbi and Smith (2019) identify loneliness as among the main sources of stress and anxiety among international students.

## Conclusion

In this paper, we have sought to make two key points. First, the data presented highlights the differentiation between international students in terms of their experiences in the host country, thus contributing to a fuller recognition of the socio-economic dimensions of international student mobility. This novel approach to understanding the experiences of student migrants contributes to contesting the still prevailing framing of the international student migrant as a privileged, discerning consumer of a service export (Gilmartin et al., 2021; Lipura & Collins, 2020; Raghuram et al., 2020). More broadly, this paper serves to nuance the association of mobility with social privilege and freedom of choice, highlighting how mobility even among relatively privileged members of the middle-class has the potential to actively contribute to the production of precarity and reduced agency. The data presented in this paper shows marked differences in students’ experiences of work and housing precarity, according to the level of financial stress reported. In effect, we demonstrate that there exists a hierarchy of privilege and risk among international students in Australia. While around 40% live without financial stress and are less likely as a result to experience anxiety about work and housing, a significant proportion of students exist at the margins, often struggling to cover housing costs, living in uncomfortable and overcrowded conditions, and vulnerable to exploitation in the workplace.

Second, we sought to move beyond both the discussion of precarity in the economic domain among international students in Australia (Berg & Farbenblum, 2019; Wilson et al., 2022), and the use of the term to denote a broad, imprecise ontological category in studies of international student mobility (Chacko, 2021; Gilmartin et al., 2021). To achieve this, the article forwards the use of the concept as a ‘relational nexus’ that links ‘questions of political economy to matters of culture, subjectivity, and experience’ (Neilson, 2019, p. 571). In this vein, we sought to operate across the divide, focusing not only on precarity in work (e.g. Standing, 2018) and housing precarity (Dotsey & Chiodelli, 2021) but also on exploring the broader implications of financial precarity for producing precariousness (Lorey, 2015) in the intimate spheres of life, taking free time, wellbeing, and relationships as three interrelated examples of mechanisms through which this occurs. The study is the first of its kind to explore these linkages. This framework could be usefully employed in future research on international students and other transient migrants.

The findings emphasise the interlinked and mutually reenforcing nature of financial insecurity and precarity in the intimate spheres’ of students’ lives. The interviews highlighted a number of mechanisms through which financial precarity shapes these facets of broader wellbeing, and through which these facets of personal precariousness may become mutually reinforcing. For example, we highlighted how the need to work and housing affordability stress contribute to time poverty, anxiety, and difficulties developing close friendships among the most precarious students. Further to this, we explored the ways in which these forms of precarity create a vicious circle wherein, as an example, a lack of free time may negatively impact wellbeing, which in turn may exacerbate financial precarity. Another concerning finding was around the physical and psychological wellbeing of international students, particularly the most financially insecure. Forbes-Mewett (2019) highlights that there are no existing government policies supporting education providers to construct adequate support mechanisms for students with poor mental health. Our study demonstrates that the most financially insecure, often the most vulnerable to these issues, are often ‘priced out’ of seeking the care they require. This highlights the need for targeted support from government for the most financially insecure international students to access healthcare services.

Through this work, we have sought to develop a deeper understanding of how financial precarity affects international students, and also to build on, clarify, and sharpen the use of precarity as a concept through which to explore their migration experiences. To do so, we adopted a framing of precarity as grounded in economic conditions, but which also speaks to the destabilising effects that precarious finances have on the broader lived experiences of international students, pointing to three specific domains of lived experience. We suggest that this framing, with a focus on the relationship between financial precarity and time poverty, personal relationships, and wellbeing, has potential for the generation of further insights into the multiple, variegated ways in which financial precarity shapes the lives of international students.

## Data Availability

All data is publicly available.

## Code availability

n/a
